# “*Pictures helped me understand it in a way words couldn’t*”: Youth reflections participating in a youth-led photovoice study

**DOI:** 10.1371/journal.pone.0308165

**Published:** 2024-09-06

**Authors:** Shelby Mckee, Tanya Halsall, Natasha Y. Sheikhan, Rodney Knight, Jo Henderson, Lisa D. Hawke

**Affiliations:** 1 Centre for Addiction and Mental Health, Toronto, Ontario, Canada; 2 University of Ottawa Institute of Mental Health Research, Ottawa, Canada; 3 University of Toronto, Toronto, Ontario, Canada; 4 Université de Montréal, Montréal, Québec, Canada; University of Saskatchewan, CANADA

## Abstract

**Introduction:**

People with lived experience of a health issue can be engaged in research to address issues related to social justice, informing change through partnerships and an understanding of community perspectives and needs. Although photovoice has been applied to various disciplines and topics across the health sciences, the concrete design of the photovoice process and participants’ experience of engaging in photovoice is not always well documented or understood.

**Objective:**

This paper describes youth participants’ experiences and perspectives with a youth-led photovoice design process on a study regarding COVID-19 vaccine confidence.

**Method:**

The sample consisted of 27 youth aged 14–24 who reported experiencing mental health and/or substance use challenges [MHSU] during the COVID-19 pandemic and some degree of COVID-19 vaccine confidence. Youth participated in a series of photography workshops, then each attended one of the six focus groups about both the topic and experience of the photovoice project.

**Results:**

Four themes were constructed from the data: 1) Participating in a photovoice project was an enjoyable experience that had a positive effect on participants; 2) Shared group experiences contributed to building a safe space for participants; 3) Photography and the photovoice process served as a catalyst for reflection; 4) Photovoice shifted participants’ perspectives on both the COVID-19 vaccine and photography.

**Conclusions:**

This project, a youth-engaged and youth-led photovoice study, describes how the photovoice methodology can be applied in a public health context to meaningfully involve young people and impact their lives. By involving youth in the co-construction of the study design and implementation, photovoice research can represent positive and empowering experiences for participants. Bringing together a diverse and multifaceted lived experience engagement research team structure strengthened the design, delivery, analysis, and interpretation of the project.

## Introduction

The engagement of people with lived experience in research has gained increased interest in health research, as practices have shifted towards addressing issues related to social justice and informing change by developing partnerships and understanding community perspectives and needs [1]. While traditional research primarily includes individuals as research participants, lived experience engagement practices in health research seek to include individuals with direct experience of a health condition in the research process as partners [2]. People with lived experience of a health condition under study can be engaged in a wide range of research designs, including qualitative and quantitative methods, and research topics that strive for concrete change or action [3,4].

Recently, there have been increasing calls for youth to be a part of the development and implementation of research conducted about them [5,6], specifically during the COVID-19 pandemic [7]. Youth can be engaged in research through a variety of methods, including participatory action research (PAR), community-based participatory research designs (CBPR), and youth-led research designs [8]. Creating partnerships in research can provide mutual benefits, increasing the validity and feasibility of the research, and helping the youth develop skills, engage socially, and gain a sense of empowerment [2,9,10].

Photovoice is a PAR methodology that engages individuals in an examination of social conditions, personal perspectives, and inequities, with the goal of advancing social justice and health [11]. Photovoice draws on the importance of individual voice and the potential for documentary photography to create social change [12]. The central aspects of photovoice are: 1) allowing people to record and reflect on issues relevant to them and/or within their community, 2) using photographs in group discussions to elicit dialogue and increase knowledge about issues, and 3) using knowledge and photographs to reach the public and policymakers [13]. Photovoice stimulates dialogue and storytelling. It provides participants with the space to observe, reflect, discuss, and communicate the strengths and weaknesses that exist within their community [13–15].

Photovoice has been used in various disciplines focused on understanding historically misunderstood settings and people with various conditions and circumstances by promoting empowerment and providing them a space to share their voice [11–13]. Photovoice has been successful in a diverse number of populations, from Indigenous communities [16,17] and chronic illness [18], to the impacts of the COVID-19 pandemic [19,20] and both mental and public health [15,21]. In recent years, this method has been implemented with engaging youth on various topics [22,23]. Youth are at a key stage in life, where experiences and environments have the potential to affect their development [24,25]. Despite this, youth voices have historically been excluded from research despite their ability to contribute [26]. Developing youth skills and encouraging them to become active members of their communities can serve to empower them with tools and knowledge towards creating positive change [27].

Although photovoice has been applied to various disciplines and topics, the concrete design of the photovoice process and the experience of the process from the vantage point of the participants is not well understood [11,28,29]. Many photovoice projects provide minimal information regarding its methods of engaging with participants [22,30]. Those that report detailed description of photovoice methods often fail to provide concrete examples that allow for replicability [23]. The literature regularly notes that participation in photovoice projects is empowering [12,15]. However, if explicit examples are not provided, this can obscure or misrepresent participants’ experience [11,28]. Addressing the ambiguity seen within photovoice with increased transparency in reporting the practice, description, and dissemination, while highlighting participants’ voices could increase the understanding of the methods, improve reciprocity within the practice, and ensure equal benefit to participants and researchers.

We conducted a youth-led photovoice project to examine the perspectives of the COVID-19 vaccine in youth with mental and/or substance use health challenges. This study was inspired by previous research that suggested the pandemic had a negative impact on youth mental health [31]. This project applied the McCain Model of Youth Engagement [8] and is in line with the Strategy for Patient-Oriented Research (SPOR) [6]. The primary findings of COVID-19 vaccine perspectives are presented elsewhere [under review]. This paper describes the youth-led photovoice design process and the related experience of participants taking part in the study.

## Method

### Study design and development

Although the funding application was researcher-led with youth input at the consultation level, upon the receipt of funding the study became led by a youth with mental health experience, with the researchers providing support for the lived experience group [32].

Lived experience engagement is described in Table 1 using a checklist for reporting on engagement in research [33]. Contributors were considered people with lived experience if they personally identified as having experienced mental health and/or substance use challenges that informed their perspectives on the project. -The structure of lived experience engagement included: 1) a youth lead research staff with a bachelor’s level of academic training, 2) a youth advisory group of 4–6 members, initially supported by two youth engagement specialists, 3) an adult photographer with expertise in photovoice and lived experience. This structure ensured that a wide variety of lived experience voices guided the project in a fulsome manner. We have outlined the lived experience roles, responsibilities, and voices in Table 2. Advisors were recruited through the institutional youth advisory network. Advisors were not participants in the study. During the planning and design phase of the project between April 2022 and March 2023, approximately 30 meetings were held that involved engagement with youth, the photographer, and other Centre for Addiction and Mental Health (CAMH) stakeholders, in addition to semi-weekly meeting with the scientific lead. For advisors and participants, we considered lived experience of mental health and/or substance use challenges together given the very high rates of comorbidity and the increasing trend toward examining such conditions together in youth research [34]. Power dynamics were managed by ensuring that lived experience voices dominated the project design and operations at various levels of engagement; ensuring open and honest discussions about challenges, questions, and progress; and acknowledging that team members held different roles, but that each had essential expertise to bring to the project.

**Table 1:** Guidance for Reporting Involvement of Patients and the Public (GRIPP2) reporting checklist for lived experience engagement in research.

Section & topic	Description
1: Aim	This study was youth-led, applying strategies for youth engagement in research, and ensured youth voices guided the project’s development, implementation of the study, and writing of the results, following a patient-oriented research approach. This study describes the process of results of a youth-led photovoice design process and the resulting experience of participants taking part in the study.
2: Methods	After an investigator-led funding acquisition stage, the study activities were led by a youth RA with lived experience, with the support of a scientist lead. During the design and recruitment stages, the youth RA was supported by 2 youth engagement specialists and a 4 member youth advisory group. During the data analysis stage, a new 6 member advisory group joined the project. A lived experience photographer was also involved throughout the project. Study participants were not involved in decision-making about the study.
3: Study results	After we received funding, the project was driven by youth voices. Study procedures, recruitment materials, and plans, development of workshops and focus groups, the interpretation and presentation of results, and the creation and plan for our knowledge translation events were all guided by youth. Youth were particularly important in guiding and making key decisions during the interpretation of our results. Researchers supported youth through methodological, analytical, and operational processes. Our lived experience photographer reinforced the quality of the participant photography and workshop content.
4: Discussion and conclusions	We were able to ensure that lived experience contributions shaped all aspects of the project. A challenge we encountered during this project was the turnover of our youth advisory group. To ensure continued youth engagement throughout the project, we were required to refresh our advisory membership. Another challenge we encountered was role confusion between our youth RA and youth engagement specialists. Given a youth RA being in a leadership position was a new structure and youth leadership was a key component of our project, it required adjustment to ensure our project’s success.
5: Reflections/ critical perspective	This project was a success due to our high levels of youth and lived experience engagement throughout every stage of the project. Youth-relevant content was delivered using co-facilitation and co-design, which is reflected in the high levels of participant involvement and positive experiences.

**Table 2:** Description of lived experience contributions and the voices brought to the study.

Role	Description	Responsibilities	Contributions to study
Youth Research Analyst (RA)	A young person with lived experience was hired in a research assistant/analyst role.	Facilitated YAG meetings, focus groups, analyzing data, and drafting the manuscripts.	Ensured youth voice in all research operations.
Youth Advisory Group (YAG)	A group that involved a total of 9 youth with lived experience was recruited to provide ongoing study guidance and feedback.	Met 15 times to provide guidance and feedback on the study during data collection, analysis, and knowledge translation.	Ensured diverse youth perspectives informed the study and increased study’s relevance to youth.
Lived Experience Photographer	An adult with lived experience and photovoice expertise supported the project.	Assisted in development of workshops, co-facilitated workshops, met regularly with the youth RA.	Ensured that photovoice training came from a lived experience perspective.
Youth Participants	Youth with lived experience participated in the photovoice study (n = 27).	Attended workshops, generated and shared photographs, and participated in focus groups.	Ensured that study data represented their personal experience with COVID-19 vaccine confidence.

**Fig 1:**
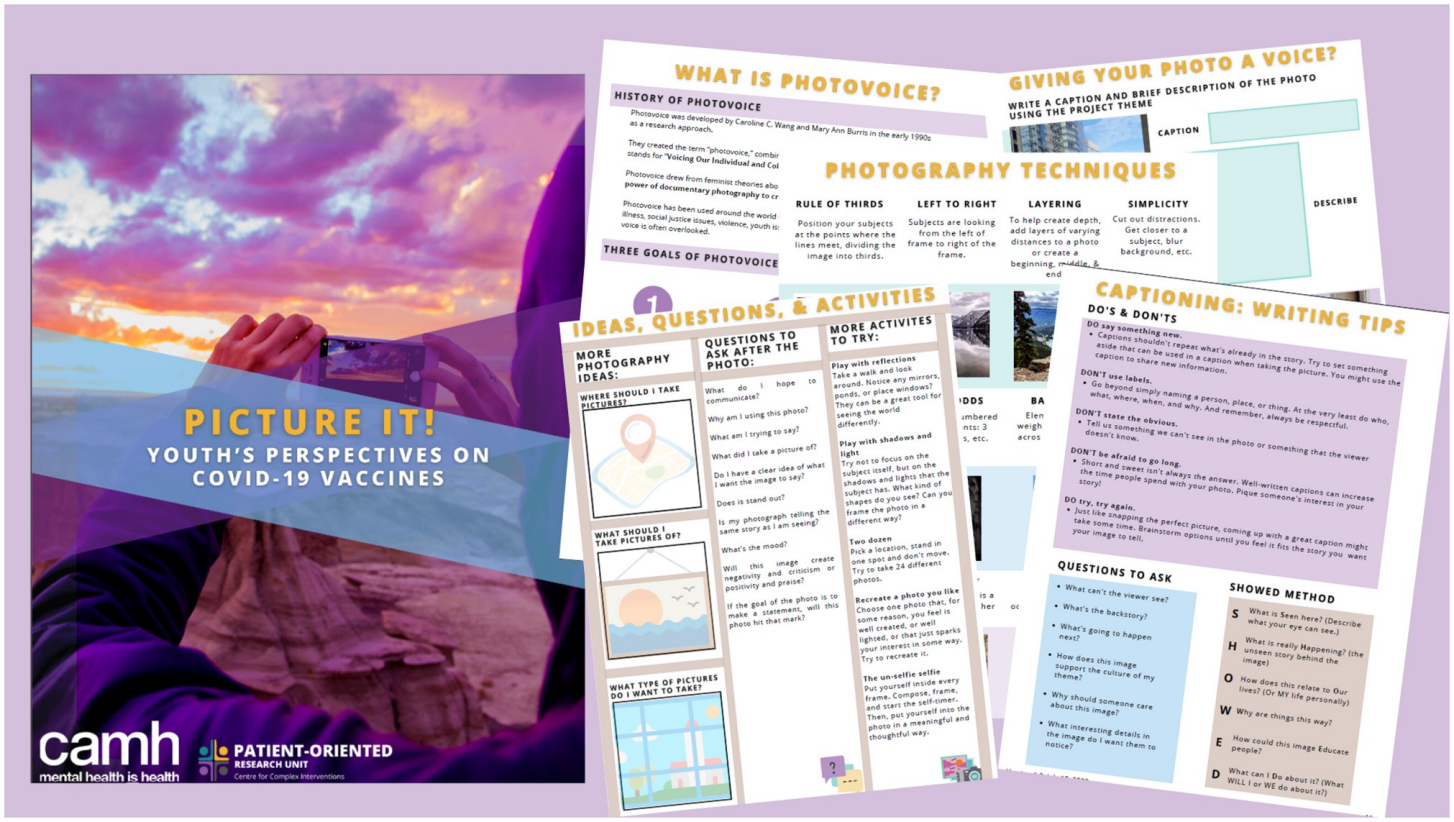
Participant photography workbook.

People with lived experience designed the project components, which included the photovoice workshops, a participant workbook, recruitment materials and graphic designs, study operational documents such as participant trackers, and the focus group guidebook. The lived experience team also co-designed multiple knowledge translation products, described below. Workshops focused on using photography to express perspectives to encourage picture-taking through activities and examples of storytelling photography. We formulated workshop activities to be relevant to abstract topics related to participants COVID-19 vaccine perspective, *e*.*g*., participants were encouraged to participate in each week’s activity and share at the next workshop. Participants used their cellphones or were provided with a camera to take photographs. The workshops built on one another and assisted participants in creating photographs, captions, and narratives regarding participants’ vaccine perspectives. Participants submitted 228 photographs throughout the workshops, with 58 final photographs submitted for the focus groups. The photography workbook provided participants with information about photovoice, the project, photography techniques, and tips for writing photograph narratives, such as using the SHOWeD method, which is typically used in photovoice projects to facilitate photography narratives. SHOWeD stands for: What do you **S**ee here? What’s really **H**appening? How does this relate to **O**ur lives? Why does the problem or strength **e**xist? What can we **D**o about this? [35,36]. Images from the photography workbook are presented in Fig 1.

### Participants and recruitment

The sample consisted of 27 youth aged 14–24 who reported experiencing mental health and/or substance use (MHSU) challenges and some degree of vaccine hesitancy during the pandemic, with the ability to hold productive conversations about building vaccine confidence. The original study from which these data are drawn from focused on the COVID-19 vaccine confidence of vulnerable youth with mental health and/or substance use challenges. Data showed that the COVID-19 pandemic was associated with MHSU challenges among youth [31]. In addition, emerging data suggested a relationship between vaccine perspectives and MHSU challenges, with higher levels of vaccine hesitancy among those with MHSU concerns [37]. This study therefore looked at vaccine confidence among this vulnerable population. Exploring whether the methodology is acceptable and youth-friendly among a vulnerable subpopulation will advance confidence in its broad applications.

Recruitment utilized multiple sources: 1) posting on social media; 2) preexisting research databases 3) outreach to professional contacts at youth organizations. Recruitment materials focused on vaccine confidence, as opposed to vaccine hesitancy, to ensure that participants were open to discussing vaccines in productive ways. Youth had to self-report some degree of COVID-19 vaccine hesitancy, presently or in the past, but an openness to productive discussion. Recruitment ran from September 15, 2022 to March 17, 2023. To support safety, we provided participants with a list of available mental health resources across Canada that they could use if they experienced distress. We further collected emergency contact information for all youth, although we did not have to use this at any time. We also checked in and debriefed with participants outside of meetings if necessary to ensure safety. Moreover, we had a clinical back-up person available if needed, although this was not needed during the study, and developed standardized work procedures to manage distress if it arose.

### Procedure

The study was approved by the CAMH Research Ethics Board (approval no. #155–2021) and was hosted by CAMH, a tertiary care hospital in Toronto, Canada. Research activities were completed virtually. Electronic written informed consent was provided during a discussion on the teleconference software WebEx. Informed consent was obtained on REDCap electronic data capture software [38]. All participants completed consent rather than assent, without parental consent, as per our Research Ethics Board approval. We completed three rounds of seven workshops between January and May 2023, virtually via WebEx. Workshops are described in Table 3. After completing the workshops, each group of participants were split into two groups and attended one 2-hour virtual focus group, for a total of six focus groups across the 27 participants. All data included within this manuscript were collected during the post-workshop 2-hour focus groups.

**Table 3:** Summary of workshops.

Workshops	Main Topic	Subtopics	At-Home Activity
Workshop 1	Introduction to the project (27 attendees)	Getting to know the research staff. Group guidelines. Getting to know participants and their goals with the project. Reviewing photography and introduction to learning to tell stories with photos.	Walk around and pretend to take pictures, what would you have captured? Or Something about where you live that you have never noticed before.
Workshop 2	Photography ethics (28 attendees, 6 photos submitted)	Last week’s activity. Photography ethics change. What is false light & how to avoid it. Consent is subjective, contextual, and circumstantial. Image theft and replication. Review the project’s subject and image release form. Example of ethics using photographer photos.	Show us “home” in no more than 3 photographs. What does home look, feel, and represent to you? Or What inspires you? What things do you find inspiring?
Workshop 3	Photography skills (26 attendees, 37 photos submitted)	Last week’s activity. Styles of photography from photographers and important features in photography. We reviewed: light, shape, contrast, color, pattern, texture, point of view, movement/motion, and focus. Example of photographer skills using photographer photos.	“Home” again.
Workshop 4	Photo Editing (23 attendees, 32 photos submitted)	Last week’s activity. Photographers who utilize photo editing. Discuss knowledge translation events. Example of photo editing using photographers’ photos.	Something that makes you feel safe or unsafe.
Workshop 5	Photo sharing (24 attendees, 36 photos submitted)	Last week’s activity. Photographers who use pictures to tell a story.	Something that makes you feel trust or distrust/comfort or uncomfortable. Or Go out and take photos when you don’t feel like it.
Workshop 6	Annotating & sharing (23 attendees, 44 photos submitted)	Last week’s activity. Photographers who use pictures to tell a story. How are you going to tell your story? Updates on the project.	Work on final project photos [COVID-19 vaccine perspective].
Workshop 7	Final share (24 attendees, 39 photos submitted)	Share final photos before focus groups. Help participants finalize project photos. Discuss final details about the project.	Finish and/or submit final photos on COVID-19 vaccine perspective.

The workshops and focus groups were co-facilitated by two members, among the youth lead, another individual with lived experience, and another research staff. Each participant was asked to come to the focus group with up to three photographs with captions and narratives; they were further asked to be prepared to discuss their photos and answer questions about their vaccine perspectives and their experiences with the project. Participants completed image release forms granting the right to use their photographs in study products, as well subject release forms if their photographs included pictures involving other people (S1 Appendix. Participant choice over the voice they expressed was ensured throughout the design of the project. Participants underwent seven progressive workshops and produced a large number of photographs over the course of the project. The data herein is drawn from the focus groups discussions; participants were instructed to select the most meaningful photos to them to bring to the focus groups, ensuring that the voice they wished to bring to the project was highlighted [11].

### Measures

Demographic characteristics were collected on REDCap [38]. Characteristics collected included general information such as gender identity, age, region of residence, and ethnic background. We also collected information on vaccine-specific variables such as vaccination status. The semi-structured interview guide was co-created by the youth-lead, our youth advisory group, and other research staff to elicit conversations about participants’ vaccine perspectives using their photography, as well as their experience of participating in a photovoice project. Focus groups were audio recorded and transcribed by a transcription agency. Transcripts were then reviewed and de-identified by the youth RA.

### Data analysis

Using reflexive thematic analysis [39], focus groups were inductively analyzed in NVivo 14 [40]. Thematic analysis is a recursive process that involves multiple stages: 1) orient to the data; 2) develop codes; 3) construct initial themes; 4) review and revise themes; 5) name and define themes; 6) write up. The analysis was conducted by the youth RA, with ongoing feedback from the research team and youth advisory group to enhance rigor and trustworthiness of the data. The present analysis centered on the questions that participants answered regarding their experience with the project. A detailed analysis of the vaccine confidence components of the transcripts is available elsewhere (under review).

### Knowledge translation

While an initial knowledge translation plan was developed by researchers, with youth consultation at the grant stage, this plan was substantially reviewed and revised by the lived experience team to ensure relevance to youth. We created a webpage to share participants’ photography and narratives with the public [www.camh.ca/photovoice]. A photobook that replicates the website was gifted to participants. We featured select photos on social media to drive traffic to the website. We sent out emails sharing our website, posted it on multiple social media channels, and posted it in a youth opportunities chat through the CAMH youth engagement initiative. We held a virtual photography event for participants to discuss and share their photography and stories with other community members. The project was featured on specialized photovoice web pages. Multiple academic posters were presented at a local and national conferences. Two academic manuscripts were also youth led.

## Results

Participants in this section were relatively diverse across many factors, including ethnicity, geographic location, and race. The majority of participants were young girls/young women (81.5%), from central Canada (Ontario and Quebec) (51.9%), and aged 18–24 (63%), with an average age of 19.6 years (SD = 3.1). All participants who finished the workshops attended one of the six focus groups. Participants attended an average of 6.3 workshops (SD = 0.9, range = 4–7).

Four themes were constructed related to participant perspectives on the photovoice process. These included: 1) Participating in a photovoice project was an enjoyable experience that had a positive effect on participants; 2) Shared group experiences contributed to building a safe space for participants; 3) Photography and the photovoice process served as a catalyst for reflection; 4) Photovoice shifted participants’ perspectives on both the COVID-19 vaccine and photography.

### 1. Participating in a photovoice project was an enjoyable experience that had a positive effect on participants

Many of our participants had never participated in or heard of the photovoice methodology before this project. One participant stated, *“I never knew about the method of photovoice before this*, *and I think it’s a cool methodology*.*” [Participant 1]*. Participants discussed how they thought it was “*a really good idea to make it like a Photovoice project*.*” [Participant 2]*. Participants expanded on this, discussing how photography is “(*a*) *powerful way to convey the moment and the impact [it can have] without having to say a lot of things” [Participant 2]* and *“sometimes experiences can’t be explained through words and as many have said*, *pictures can be worth 1000 words*.*” [Participant 3]*.

Participants discussed how they enjoyed that “*pictures are used in this project to express our feelings*.” *[Participant 4]*. Many felt the use of photography in this project, specifically in the focus groups, helped them express themselves and tell their story:

“*I think photography is a wonderful way of showing a story*. *Because a photo can say so much where… with words*, *you’re kind of limited*. *I mean*, *like earlier*, *I got so much anxiety*. *I didn’t know what I wanted to say*. *But I feel like my photo–obviously because I took it–I knew what it means… I felt represented everything that I wanted to say*, *where maybe I couldn’t find the words*.”

*[Participant 5]*.

Participants also discussed enjoying how the workshops were designed, stating, “*the theme words were helpful…and how they related to the vaccine and how I could portray that in my photos” [Participant 1]*. They expressed that the overall atmosphere of the workshops felt like everyone wanted to be a part of the project. Participants were thankful for what they considered a special experience; they enjoyed learning about and seeing other’s photography and perspectives:

*“I think it’s really special that everybody who is here*, *kind of*, *I feel like*, *they want to be here and they want to tell their stories and they want to have their voices heard*.*”*

*[Participant 2]*.

“*Thank you*. *It was a great learning experience*. *The different pictures were fun to look at and learning everyone’s experience is really great*. *Thank you for teaching us*.*”*

*[Participant 6]*.

### 2. Shared group experiences contributed to building a safe space for participants

In the safe space created for the project, participants were able to discuss their COVID-19 vaccine experiences while embracing the similarities and differences of other youth experiences. Participants discussed the differences between photovoice and other types of research projects, emphasizing the value of having multiple meetings with the same group of people. Participants believed that this allowed everyone time to become more comfortable sharing their stories and perspectives with others. Multiple meetings created group cohesion and a sense of safety:

“*We’ve been working together*, *the group*, *for two months*, *and that’s why I felt like–I think that’s why everybody felt comfortable to share today… Some of the projects*, *they have one meeting and then they ask you a bunch of questions on that one meeting and then you just don’t feel comfortable enough to share*.*” [Participant 2]*.

Many participants discussed how group meetings became a “safe space” for youth to talk about the COVID-19 vaccine and aspects of the pandemic. For instance, some highlighted that in the group meetings created “*a very inclusive accepting space for us to bridge and delve into the sensitive topics*.” *[Participant 7]*. Participants believed that the atmosphere created was a group effort by both the facilitators and participants. Furthermore, they discussed how they were not talking about the COVID-19 vaccine in their lives because it is a “taboo topic” and it was nice to have a space to be able to do that. For example:

*“It’s been nice to sort of see everyone else’s perspectives on the pandemic*, *because a lot of the time vaccines or COVID are very*, *taboo topics*. *You don’t really bring it up in a conversation at like dinner*, *because it’s very*, *touchy*. *So this was kind of nice to have a safe space and see where everyone’s coming from and see the different experiences that we all had*.*”*

*[Participant 8]*.

Participants described that by *“learning about others experiences of COVID” [Participant 9]* they “*gained a lot of knowledge in these past couple of weeks of participating in these workshops so I’m really grateful*.*” [Participant 10]*. Participants highlighted that hearing others’ stories made them realize that although everyone has their unique stories, people often experience similar thoughts and feelings. For some, this made them feel less alone and realize the importance of having these conversations:

“*It definitely allows us to see how someone or how anyone*, *no matter how*, *who they are*, *or where they come from*, *or what their background is*, *truly might experience the same things as us*, *especially over the past few years*. *And just really impactful to see how we all have a piece to play and how we all have that*, *how we are all impacted over the past three years*.”

*[Participant 7]*.

For other participants, having the opportunity to listen to people’s stories and perspectives allowed them to empathize and relate more because they were able to better understand where they were coming from:

“*All of the layers of stuff before that are like what’s making people make the decision they make*. *And it just makes you be able to empathize with people who have a different perspective than you*, *or understand why they have that perspective more*. *But that’s really cool to be able to do that and just feel like more relating to people who maybe have different perspectives or different positions…and be able to better understand where they’re coming from*.*”*

*[Participant 11]*

### 3. Photography and the photovoice process served as a catalyst for reflection

While talking about the process of the project, participants discussed how photovoice is a “*means for reflection*.” It provided them with an opportunity to reflect on their perspectives and experiences with the COVID-19 vaccine and the pandemic. For example, one participant stated that “*Photovoice has provided a reason*, *or excuse to think more about the vaccine and how I felt*. *It’s not really something I would have thought about normally*.” *[Participant 12]*. Similarly, another participants felt “*it was just a cool*, *fun way of*, *I guess explaining myself*, *is what I saw it as*. *And yes*, *it definitely did make me reflect on it*.*” [Participant 13]*.

Participants went into detail about reflecting on and revisiting their experiences and choices with the COVID-19 vaccine. For example, on participant stated: “*It really made me reflect on a lot of the choices that I made*. *And maybe I should have done this and this*. [*…] it just made me question a lot of things that I did*.*” [Participant 5]*.

Other participants described that the act of reflection allowed them to confront their experiences with a different frame of mind and work through unprocessed emotions:

“*I think for me*, *there was a lot of unprocessed emotions that I had from this time*. *So actually*, *sort of making space for me to process everything*, *was uncomfortable at first because again*, *like [participant name] said*, *it was kind of*, *we’re going back in time*. *I don’t necessarily want to go back to where I was or that headspace*. *But then also*, *I think*, *in this experience*, *it’s been nice to sort of see everyone else’s perspectives on the pandemic*.”

*[Participant 8]*.

Participants also discussed that the process of taking photographs made them address their emotions and helped them better understand their experiences with the vaccine:

“*I don’t think I really like thought much about the vaccine before*. *I mean I only really thought about how like–it was a bit like scary being pressured to get it*. *But also like the different opinions and stuff like that*. *But I feel like being able to take pictures also helped me like explain even to myself how I felt*. *Yeah*, *because like just looking at the pictures and like trying to connect it to how I feel*, *it helped me like understand it more*. *Like with not the words could*. *I don’t know how to word it but it’s like the pictures helped me understand it in a way words couldn’t*.*”*

*[Participant 14]*.

### 4. Photovoice shifted participants’ perspectives on both the COVID-19 vaccine and photography

Participants talked about the learning process they experienced throughout the course of the project and how this impacted their perspective of the COVID-19 vaccine, photography, and diverse experiences. The project led them to think about their thoughts and feelings towards the COVID-19 vaccine from a different perspective. One participant found that the reflection process “*was definitely difficult to go back into that headspace to think about COVID when I just wanted to be done with it” [Participant 5]* but through this process *“it did change my perspective*.*” [Participant 5]*.

Having to express their vaccine perspective through photography *“can be so therapeutic” [Participant 1]*, while helping them make sense of and work through their thoughts and emotions towards the vaccine, while having *“a lot of mixed emotions and feelings toward it*.*” [Participant 15]*. This experience enabled the same participant to become more aware of what they felt towards the vaccine, stating “*my feeling towards the vaccine grew stronger… so the workshop[s] was really good for that*.*” [Participant 15]*.

However, some participants did not experience a change of perspective on the vaccine throughout this project: *“I don’t think it’s changed my feelings on vaccines for me personally*, *but it’s definitely made me feel like excited by that potential to talk about them*.*” [Participant 11]*.

Participants discussed the value of having a creative space to be open and honest regarding their feelings and experiences. Participants described that it was *“kind of a healing process in a way” [Participant 11]* and how hearing others talk about their feelings and experiences through “*the project helped me to open my mind to different mindsets*.*” [Participant 16]*. Participants enjoyed the opportunity to talk about the vaccines so much so they “*wish there was more ways and more like platforms where that was happening*.*” [Participant 11]*.

Participants also discussed how the group setting challenged their perspective by giving them the time and space to begin to understand how others felt:

“*So I really needed to take some time to understand how other people felt about it*. *Which is why I said about that photo*, *is like changing perspectives because I really needed to challenge my perspective*. *Not only when I was taking the photo but when I was having discussions about other people about the vaccines*.”

*[Participant 17]*.

Participant photo. "I really needed to challenge my perspective. [I]t’s easy for me to say, “Well, I’m going to get the vaccine and you do whatever you want.” But it takes another level of emotional maturity to understand why."

Participants discussed that through this project, their view of photography changed and that they learned how to use photography to tell stories and share a message with others:

“*Before this workshop […] I wasn’t really interested in using photography to tell a story*. *I was simply just trying to use photography to capture moments*, *create photo books*, *create those memory videos*, *just for me to kind of relive and reminisce in those times*. *But throughout this project*, *I definitely learned how to utilize photography and photos to tell a story and to help reinforce a message*, *a very positive message*.”

*[Participant 7]*.

Participants described that the workshop activities made them more present and mindful when going out and searching for things to photograph. Participants discussed how this project changed how they looked and interacted with the world, with one participant stating, “*I never had to look at the world in that specific way*.” *[Participant 18]*. Another participant discussed *“topics weren’t easy to find*. *It wasn’t something simple like taking a picture with the color purple*. *Instead there had to be a story behind [it] and what we felt personally*.*” [Participant 14]*. Through changing the way they looked at the world, this participant began taking pictures of things they never would have before, stating, “*I never would’ve really thought of taking a picture of it*.*”[Participant 14]*. Other participants discussed having to *“make them [photographs] interesting instead of just seeing them*, *thinking they were interesting and capturing them in a way that was interesting for others to view*.*” [Participant 6]*.

Finally, one participant discussed if they were to summarize this project, it would be that youth experienced “*The intense change*. *I think maybe not just looking at the photos*, *but hearing all of our stories together*, *they would see that all of us experienced change*. *And as adolescents*, *that’s a lot harder to go through*.*” [Participant 5]*

## Discussion

This study examined the process of the youth-led photovoice design and the resulting experience of participants taking part in the study about youth perspectives on COVID-19 vaccine confidence. Four themes were constructed: 1) Participating in a photovoice project was an enjoyable experience that had a positive effect on participants; 2) Shared group experiences contributed to building a safe space for participants; 3) Photography served as a catalyst for reflection; 4) Photovoice shifted participants’ perspectives on both the COVID-19 vaccine and photography. Participating in a youth- and lived experience-led photovoice study was a positive and empowering experience for participants.

It is recognized in the literature that the success of photovoice is the ability to encourage and support participants to use photography as a means of expression by creating a space that allows for personal exploration, critical thinking, self-representation, and community action [29]. However, there is a lack of consistency in project design, methods, and reporting of photovoice projects [28]. Much of the research focuses on the results of the photovoice project or the topic of interest and its effectiveness for the studied population, but do not include the experience of the project through participants’ voices [22,23,30]. Our project sought to fill this gap by directly highlighting the experience of youth participating in a photovoice project in their own words.

Participating in this study and engaging with photography provided participants with opportunities to not only reflect on the topic at hand, but also develop new skills and perspectives. As highlighted by our participants, photographs can be used as mechanisms to assist and encourage deeper reflection on lived experience and facilitate richer personal narratives [28]. Findings are in line with previous research on the way images affect individuals, particularly youth. Taking pictures and reflecting on events have been shown to have a therapeutic effect through garnering self-enlightenment and/or new perspectives [15,41]. This may be due certain forms of memory recall being inherently image-based [42]; it has been proposed that images may be able to penetrate the consciousness into aspects of an individual’s experiences that words cannot [43,44]. Similarly, creating images gives youth opportunities for new forms of perception and relational thinking [45]. When youth learn to critically analyze social environments through engaged activities, they do not internalize oppressive sociocultural influences in the same way [46]. Enhanced critical thinking might be an important way to help young people reflect on misinformation and critically assess information circulating in the public. Photovoice therefore appears to be a research methodology that leverages the power of images to support youth in a critical thinking process.

Research has shown that photovoice projects often lead to an improved understanding of the community, enhanced engagement within the community, and increased individual empowerment [21,47]. Indeed, participants in the current study spoke of the positive impacts of group experiences and the opportunity to transform their way of thinking about the COVID-19 vaccine. Empowerment may emerge as an individual learns to analyze their environment, expanding their critical thinking skills while helping them develop supportive relationships that promote collective and individual efficacy [29]. The training within photovoice can thereby be an empowering experience, as it can provide individuals with new collective and individual experiences, while opening up new possibilities for them to express themselves [28]. As noted in our project and other research, the context and group dynamics are crucial factors that support positive study outcomes and foster social skill development, feelings of community, and social connectedness [48–51].

It is especially important that photovoice research teams carefully consider how the photovoice process will be implemented to ensure they are supporting meaningful engagement throughout the entirety of the research [52]. It is critical to understand the responsibility a project has to avoid causing further harm to participants by performing tokenistic participatory research. Although facilitating social and policy change is not always a realistic goal, creating change within the community of one’s project is possible and represented in our project.

The strengths of this project include being youth led, having youth and individuals with lived experience engaged in all aspects of the project, strong retention of participant involvement, and a large and relatively diverse national sample. This study applied a lived experience engagement lens rather than a PAR lens, which appears to have been an appropriate adaptation. We implemented a number of strategies to ensure that lived experience led this study: a youth with MHSU lived experience led the work, supported by a youth advisory group and an adult photographer with MHSU lived experience. Together, these team members shaped the project and the outputs in important ways. Notably, the lived experience-led design and photovoice methodology helped honor participants’ lived experience by building a project that provided the opportunity to properly facilitate their reflection, gave them a platform, and amplified their voices in ways that other methodologies might not [28,44].

However, limitations should be kept in mind when interpreting the results. Despite being relatively diverse, some subgroups were underrepresented. Due to this, we were unable to make any conclusions regarding demographic characteristics. With this project being conducted virtually, we were able to recruit Canada wide and have high participant retention. However, this limited our sample to those who had access to a computer and internet. An additional limitation is that the data on participants’ experiences of the project were collected by the research staff member who facilitated the project, which may have led to social desirability. In addition, participants self-selected to participate in the project and may have therefore been individuals who were more likely to enjoy participating in research. Future research should connect with youth who do not have access to regular internet or computers and examine the issue with various diversity subgroups.

## Conclusion

This project is an example of a youth-engaged and youth-led photovoice project that demonstrates the success of photovoice as a methodology applied to a public health issue. Multi-facetted lived experience engagement strengthened the design, delivery, analysis, and interpretation of the project. Participants found it to be a meaningful way of deepening their perspectives. Given the broad and overarching strengths of this photovoice methodology, this study demonstrates its benefits to the communities engaged in the research.

## Supporting information

S1 Appendix Fig 1. Release form. Sample release form for the public use of photographs.
(DOCX)
