# Supplementary material for: “Pictures helped me understand it in a way words couldn’t”: Youth reflections participating in a youth-led photovoice study
Source: PLoS One. 2024 Sep 6;19(9):e0308165. doi: 10.1371/journal.pone.0308165 (PMC11379270; doi:10.1371/journal.pone.0308165)
Supplement: S1 Appendix — Release form. Sample release form for the public use of photographs. (DOCX) [file pone.0308165.s001.docx]

**Public use of Participant-Photographs and Participants first name**

Pictures taken may be shown to others in the community at large, with possibly no connection to the Centre for Addiction and Mental Health (CAMH), in gallery displays, public shows, presentations to decision makers, published on the CAMH website, and/or shared over CAMH’s social media channels. They will be used to create awareness about youth perspectives on COVID-19 vaccine hesitancy and confidence.

Are you, as a participant-photographer, willing to have your photographs used in public displays, shows, presentations and on the CAMH website in perpetuity?

Yes No

Do you agree to the use of your photo captions and descriptions in public displays, shows, presentations and on the CAMH website?

Yes No

Do you want your name listed as the participant-photographer? (First name only)

Yes No

Do you agree to the use of your still and/or moving portrait in public displays, shows, presentations and on the CAMH website?

Yes No

By signing my name below, I understand and agree that:

- I understand that CAMH will not use my photography for promotional purposes. I understand and agree that CAMH may contact me regarding the submission of photos of me and/or my likeness for this projects promotional purposes.
- I understand that by signing this document my photography may be shared to the media for editorial purposes.
- I understand that CAMH may work with the media to promote this work; in this case, by consenting to the use of my photography for promotional purposes, I understand that my photography may reside on 3rd party sites and CAMH will not have the authority to remove it.
- I understand that I have the right to withdraw my consent to the use of the items indicated above for this purpose at any time before they are used and/or distributed. If I want to withdraw my consent I will use the contact information provided below.
- I also understand that CAMH does not have to notify me when it uses or shares the items as agreed to above, and I will not be asked to approve the items listed above before they are used or shared for the purposes listed on this form.
- I hereby release CAMH and its officers, directors, employees, clinicians, agents, successors and assigns from all actions, claims and demands arising from the above consent and the collection, use, disclosure, retention and destruction of the photographs, pictures, videos and recordings as described above.
- I confirm that I have had the opportunity to discuss this activity and my consent with CAMH staff and clinicians. I understand that my decision whether to participate is completely voluntary and will not affect any care that I receive.

Name of Participant Photographer Signature DD/MM/YYYY

If you would like to contact the researchers for any reason, including your right to withdraw consent to the use of your items, you may contact:

Primary Investigator: [Lisa.Hawke@camh.ca](mailto:Lisa.Hawke@camh.ca) Phone or text: +1 (416) 518-4658

Research Analyst: [Shelby.McKee@camh.ca](mailto:Shelby.McKee@camh.ca)

**Release Form for Subject of Participant-Photograph**

You are invited to have your picture or a picture of your property taken by one of the photographers involved in a Photovoice research project at the Centre for Addiction and Mental Health (CAMH). Photovoice highlights the voices of people who might otherwise not have a say. It gives people the space to uncover, clarify, and strengthen the voice they already have using photography. This Photovoice research project focuses on the topic of COVID-19 vaccine hesitancy and confidence to raise awareness and promote discussions surrounding this topic.

Photovoice has three goals:

1. Guiding people in recording and reflecting their community's strength and concerns
2. Promoting critical dialogue and knowledge about important issues through large and small group discussions of photographs
3. Reaching the public and policy makers and encouraging policy makers to adopt informed health policies

Pictures taken for this research project may be shown to others in the community at large with possibly no connection to CAMH, in order to create awareness. This may include gallery displays, shows, presentations to decision-makers, in education videos, published on the CAMH website: [www.camh.ca](http://www.camh.ca) and/or shared over CAMH’s social media channels. Others viewing the pictures may recognize you, but there will be no full names or contact information. However, with participant-photographer permission, CAMH may choose to have first names associated with the photograph, for editorial purposes. Photographs will never be used to make money.

By signing my name below, I understand and agree that:

- Unless otherwise stated in writing by the participant-photographer, CAMH and other organizations assume that permission is granted to use my photographs for project-related reports, exhibits and presentations that are likely to result from this project.
- That researchers, policy makers, students, and possibly people from my community will see my photo.
- That photos of me and/or my likeness may be used in a public setting and displayed indefinitely.
- I understand that CAMH will not use my photograph for promotional purposes. I understand and agree that CAMH may contact me regarding the submission of photos of me and/or my likeness for this projects promotional purposes.
- I understand that by signing this document my photography may be shared to the media for editorial purposes.
- I understand that with participant-photographer permission, CAMH may choose to associate my first name with the photograph for editorial purposes.
- I understand that CAMH may work with the media to promote this work; in this case, by consenting to the use of my photography for promotional purposes, I understand that my photography may reside on 3rd party sites and CAMH will not have the authority to remove it.
- I understand that I have the right to withdraw my consent to the use of the items indicated above for this purpose at any time before they are used and/or distributed. If I want to withdraw my consent I will use the contact information provided below.
- I also understand that CAMH does not have to notify me when it uses or shares the items as agreed to above, and I will not be asked to approve the items listed above before they are used or shared for the purposes listed on this form.
- I hereby release CAMH and its officers, directors, employees, clinicians, agents, successors and assigns from all actions, claims and demands arising from the above consent and the collection, use, disclosure, retention and destruction of the photographs, pictures, videos and recordings as described above.
- I confirm that I have had the opportunity to discuss this activity and my consent with CAMH staff and clinicians. I understand that my decision whether to participate is completely voluntary and will not affect any care that I receive

Subject Name: _________________________________________

Signature: ___________________________________________

Name of Participant-Photographer: __________________________________

DD/MM/YYYY: _____________________________________

If you would like a copy of the photograph taken of you, please write down your mailing and/or email address.

______________________________________________________

If you would like to attend the researchers projects knowledge sharing activities you may contact;

Email: [photovoiceproject@camh.ca](mailto:photovoiceproject@camh.ca) Phone or text: +1 (416) 518-4658

If you would like to contact the researchers for any reason, including your right to withdraw consent to the use of your items, you may contact:

Primary Investigator: [Lisa.Hawke@camh.ca](mailto:Lisa.Hawke@camh.ca) Phone or text: +1 (416) 518-4658

Research Analyst: [Shelby.McKee@camh.ca](mailto:Shelby.McKee@camh.ca)
